# A Framework for Assessing Resilience in Urban Mobility: Incorporating Impact of Ridesharing

**DOI:** 10.3390/ijerph191710801

**Published:** 2022-08-30

**Authors:** Dawei Li, Yiping Liu, Yuchen Song, Zhenghao Ye, Dongjie Liu

**Affiliations:** 1 Jiangsu Key Laboratory of Urban ITS, Southeast University, 2 Dongnandaxue Rd, Nanjing 211189, China; 2 Jiangsu Province Collaborative Innovation Center of Modern Urban Traffic Technologies, 2 Dongnandaxue Rd, Nanjing 211189, China; 3 School of Transportation, Southeast University, 2 Dongnandaxue Rd, Nanjing 211189, China

**Keywords:** resilience, system safety, urban mobility, ridesharing, population density

## Abstract

To a certain degree, the resilience of the transportation system expresses the safety of the transportation system, because it reflects the ability of the system to maintain its function in the face of disturbance events. In the current research, the assessment of the resilience of urban mobility is attractive and challenging. Apart from this, the concept of green mobility has been popular in recent years. As a representative way of shared mobility, the implementation of ridesharing will affect the level of urban mobility resilience to a certain extent. In this paper, we use a data low-intensity method to evaluate the urban traffic resilience under the circumstance of restricted car use. In addition, we incorporate the impact of ridesharing services. The research in this paper can be regarded as an evaluation framework, which can help policy makers and relevant operators to grasp the overall resilience characteristics of cities in emergencies, identify weak sectors, and formulate the best response plan. This method has been successfully applied to two cities in China, demonstrating its potential for practice. Finally, we also explored the relationship between urban traffic resilience and the pattern of population distribution. The analysis shows that population density has an impact on the level of transportation resilience. And the incorporation of ridesharing will bring an obvious increment in resilience of most areas.

## 1. Introduction

Over the years, due to the frequent occurrence of some extreme weather conditions and the fluctuation of transportation fuel prices, various negative impacts have occurred on the urban transportation system [1,2], for instance, breakdown of the road network, increase on the cost of motor vehicle trips, decrease in the capacity of the road link, and so on. This has seriously affected the safety of transportation system operation and the negative influence of these events show that the traffic reliability on cars is fragile. Apart from this, many nations and organizations, such as the European Union [3], and China [4], are accelerating the process of reducing greenhouse gas emission, which contribute to the social and economic uncertainty in relation to urban mobility. Moreover, due to its characteristics of eco-friendliness and convenience, shared mobility has been promoted in cities. As a representative form of shared mobility, benefiting from the development of the online hailing platform, ridesharing services have developed rapidly in recent years. Considering the situation, we apply a concise framework to analyze the resilience of urban mobility under the circumstance that car trips are restricted, the specific disturbance event can be various, for instance, an energy crisis, economic unrest. This paper only focuses on the final influence of these circumstances on transportation system. In this framework, we incorporated two active modes, including cycling and walking, which would be less affected by the circumstances, and the impact of ridesharing services. Referring to study of Martins et al. [5], we introduced a data low-intensity (which means the data needed are moderate) method to assess the degree of dependence on cars of one city under the assumed circumstance, through calculating the proportion of car trips able to be converted to active modes. We improve the original methodology by considering ridesharing, which is playing an important role in daily transportation. The main purpose of the method is to help transportation managers and decision makers assess the system’s ability to maintain its function when car use is restricted and to provide suggestions for operators about making ridesharing strategies.

The concept of resilience was first introduced into the ecosystem in the early 1970s [6]. It is defined as the ability of the system to maintain the durability and stability of its relationship in the face of external disturbances, and the idea of quantifying it is preliminarily put forward. Its applications later in the transportation field are mostly used as the objective function of system optimization to evaluate the cost level [7,8]. In reality, the definition of resilience is not uniform. For example, Fisher proposed more than 70 methods to display the definition of resilience [9]. However, the definition of transportation system resilience in existing studies can be summarized as the ability to resist disturbance (robustness, vulnerability) [10,11], and the ability to restore the function after disturbance (recoverability) [12]. In this paper, the concept of resilience is defined as the ability of transportation system to maintain its function during the disturbance events. This definition can reflect the robustness and vulnerability in some degree. In our study, we mainly focus on the system’s function of satisfying people’s travel demand during the event.

In recent decades, with more appeal for greener mobility, many innovative transportation modes and techniques are promoted in daily life, such as electric vehicles and shared mobility, and many studies have been conducted on them [13,14]. Benefiting from the development of communication technologies and computer science, ridesharing, as one representative types of the shared mobility, has developed very rapidly in recent years. Ridesharing is defined as travelers with similar origin and destination or partial same path are aggregated to share the same vehicle so that they can share travel expenses equally or make a profit. From the perspective of travelers, the adoption of the ridesharing system can reduce travel costs and parking costs, while from the perspective of traffic systems, this travel mode can effectively decrease urban traffic load. As early as the late 1980s, studies showed that ridesharing accounted for a considerable share of the daily travel [15]. In China, the scale of ridesharing has also been expanded [16]. The promotion of ridesharing services is conducive to the alleviation of urban traffic congestion [17], the solution of urban parking space problems [18], and has a positive impact on the environment [19]. With the emergence of online platforms, scholars pay attention to the efficiency of different ridesharing matching strategies for taxi operators [20].

The purpose of this study is to propose a framework for combining the ridesharing service with active travel modes in the evaluation of urban traffic resilience. Our work is a new iteration of an existing study [5]. We assume the circumstance that the car trip is restricted because of natural or socio-economic events, and we successfully apply the framework to two distinctive cities in China. In this study, we stress the impact of different ridesharing service strategies. Apart from converting replaceable car trips into active modes, the hailing platform can also try different ridesharing matching strategies to decrease the car trip demand so that the negative influence can be lowered. This method could be applied with merely OD data. The work can help decision makers have a whole view of the transportation system during the event so that they can grasp the weakness of urban mobility and try to formulate an optimal traffic resources allocation scheme. In general, this can help creating management measures both in strategic and operational layers. Moreover, we have explored the relationship between the resilience results and the spatial distribution of urban population through analyzing it specifically in administrative divisions.

Next, we will briefly review the framework of resilience and associated methodologies to quantify this and the evolution of research in ridesharing. The motivations and contributions of this paper will also be illustrated.

### 1.1. Literature Review

Resilience is a definition originated from the area of civil engineering to evaluate the endurance of external force, which is then applied to the ecosystem. The conceptual framework of resilience was originally developed since 2003 [21], its main principle incorporates ‘reducing the possibility of failure’, ‘reducing the consequences of failure’, and ‘reducing the time to recover from failure’. Thenceforth, the concept was broadly applied to the field of transportation, and many scholars presented their own interpretations on the definition of transportation resilience. Ref. [22], introduced that the four aspects of transportation resilience are: latitude, resistance, precariousness, and panarchy. Ref. [23], proposed that a characteristic of resilience is the ability of the system to operate and recover under abnormal conditions. Ref. [24], defined traffic resilience as the ability of a transportation system to absorb risk, maintain function, and recover from disruptions.

Due to the frequent occurrence of extreme weather and some other social and economic unrest, resilience has been a trendy topic in the related transportation research area. Tonn et al. recently proposed research about the evaluation of transportation infrastructure resilience under the influence of social policy [25], and this is a tendency to connect resilience assessment with policy decision. Quite a few studies focus on the relationship between natural hazards and resilience [26,27]. Apart from these, some scholars focused on the social and economic level. Mattioli conducted a study about the impact of fuel price fluctuation on vulnerability [28], Buinevich forecasted the resilience of ITS under cyber-attacks [29].

Many approaches have been proposed to assess the transportation resilience, both qualitative and quantitative ones. Shekar conducted simulation with sumo to evaluate the resilience of a simple road network. The evaluation index adopted was the delay time of the vehicle [30]. Wang used an end-to-end deep learning method to assess the resilience of Shenzhen’s transportation network under extreme weather conditions [31]. The evaluation index is the integration of capacity retention ability and recovery ability of transportation network. For resilience of multi-modal transportation system, Baggag adopted the supra-Laplacian matrix to evaluate the coverage of the urban road network by coupling different modes in the transportation network [32], and thereafter obtained an evaluation of the resilience of the entire city. A method is presented that considers the conversion between active travel and passive travel, and applied OD data to calculate the proportion of trips which can be converted to active travel as an indicator to evaluate urban transportation resilience [5]. However, this method only considers the modes of cycling and walking, and we have boosted the consideration of ridesharing to this method.

Most of the literature on ridesharing focus on its impact on traffic flow or its operational layers. Some of these studies are based on an approach of constructing a mathematic model, for example, Ma constructed a stochastic ridesharing equilibrium considering elastic demand [33], and Di formed a model based on link and node to get a ridesharing equilibrium [34]. While some other researchers rely on big data, Veve categorized trips in accordance to regularity and similarity [35]. After modeling different clusters, thereby the number and location of the service demand can be predicted. Zhong and Sun analyzed travelers’ behavior when using hailing platform by applying float car data [36].

With regard to the literature related to ridesharing strategies, most of the current studies focus on the impact of different ridesharing strategies on traffic demand and travelers’ behavior. Fiedler’s research combines travelers with similar travel trajectories and limits the waiting time boundary, and measures the impact of this service on urban traffic demand [37]. Ma formulated the dynamic ridesharing charging policy from the perspective of space and time and assessed its impact [38]. Ehsani considered the weighted influence of ridesharing service detour distance and waiting service duration on travelers [39].

Based on the above research, one thing that cannot be ignored is that the mode of ridesharing is playing an increasingly important role in our transportation system, while almost no literature has paid attention to its influence on transportation resilience. The data demand of some methods is quite complicated, which means managers may not be able to get a preliminary analysis quickly during the emergency events. For this reason, the use of comparative low-intensity data to get a resilience analysis result and the incorporation of ridesharing in resilience assessment are necessary to adapt to the requirement of reality.

### 1.2. Contributions

The main purpose of this paper is to make an overall assessment of urban traffic resilience under the scene that motor vehicle use is restricted, and the available data is non-intensive. This paper proposes a framework which focuses on the impact of ridesharing services on urban traffic resilience. In view of the motivations, this paper presents the following contributions:

- Incorporating consideration of ridesharing service in a methodology to evaluate the resilience of urban transportation network, which is innovative in this area;
- Through applying the method to two different cities, we testify the feasibility of the work proposed, which can provide suggestions for related decision makers and operators;
- Exploring the relationship between regional resilience and population density.

The rest of this paper is organized as follows. Section 2 specifically introduces the methodology of the framework and the way to incorporate ridesharing services into this. Section 3 illustrates two cases of Shenzhen and Haikou, respectively, and makes comparative analysis on their behavioral and spatial features. Finally, Section 4 concludes the whole paper and outlooks the future research.

## 2. Methodology

**Figure 1:**
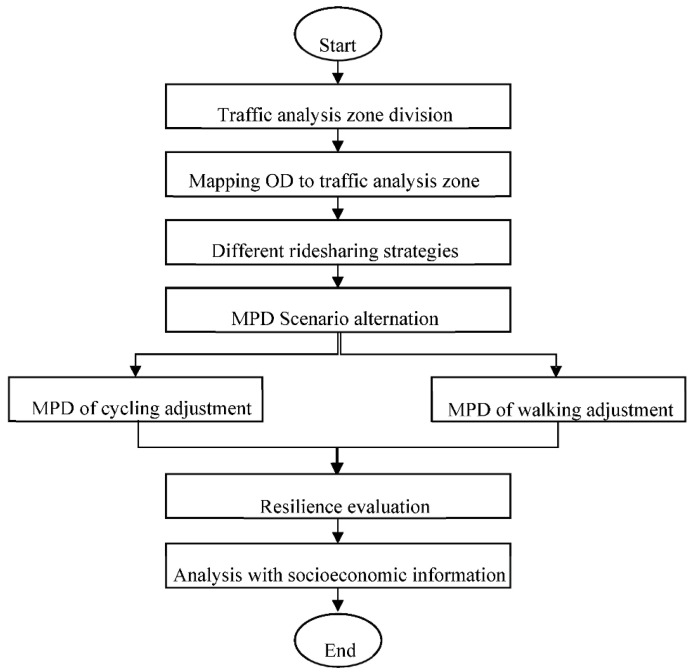
The structure of the method.

In this section, we give a detailed introduction to our methodology and provide an example to illustrate this. The structure of the method is shown as followed in Figure 1.

The original method evaluated the resilience by calculating the ratio of motorized mobility in the city which can be converted to cycling and walking when the car is restricted to use [5]. The definition of resilience in our study is the ability of the transportation system to maintain its function under specific circumstances. In our method, the negative influence due to car restriction can be decreased by two ways: ridesharing reduces car trip demands, while MPD scenarios can transfer car trips. Then we can judge that this method is reasonable in this paper, because in the scene of limited car travel, the high percentage of car trips which can be converted to non-motorways means that the transportation system can meet more individual travel needs in this case. This method establishes various scenarios by adjusting the maximum potential distance (MPD) for cycling and walking. The analysis of results explores the relationship between urban transportation resilience and MPD. Depending on this, in our augmented method, before evaluating the relationship between resilience and MPD, we first considered the possible impact of a ridesharing service through adopting various matching strategies. By means of presetting parameters of the ridesharing service, the total amount of travel is processed first. Afterwards, the proportion of trips which can be converted to an active mode under specific scenarios is calculated. This method uses urban OD travel data and divides the transportation analysis zones of the study area by rasterization to determine clusters with similar trips.

### 2.1. Quantitative Way for Resilience

The maximum possible distance (MPD) is defined as the farthest travel radius of a certain travel mode. In this paper, we use the MPD of walking and cycling to construct the scenarios. It is worth noting that considering the realistic situation, the MPD of walking should be less than that of riding. The quantitative way of the resilient level is to examine the proportion of motor vehicle travel distance within the MPD of walking and riding. In other words, when motor vehicle is limited, what proportion of these kinds of trips can be satisfied and converted to walking and cycling decides the resilient level of urban mobility, which are more active modes.

Based on the definition of [5], we complement it and the motor vehicle trips can be divided into the following categories:

- Persistent—travel distance less than MPD of walking;
- Adaptable—travel distance exceeding MPD of walking but is less than MPD of cycling;
- Transformable—travel distance exceeding MPD of cycling;
- Flexible—travel demand reduced due to ridesharing.

On the basis of the existing research, a ridesharing service can significantly reduce the amount of urban trips [40]. Consequently, in this paper what is distinctive from [5], is that we specified the reduced travel demand caused by adopting different ridesharing strategies as flexible trips. In all categories of motor vehicle travel, persistent, adaptable, and flexible trips are classified as resilient trips. The formula for calculating the resilient level is shown as followed:

$$Resilient\;level=\frac{Persistent\;trips+Adaptable\;trips+Flexible\;trips}{All\;trips}$$

The resilient level can be explained as a proportion of car trips which can be converted to active modes or decreased due to the ridesharing service in the circumstance of restricted car use. This indicator can represent the degree of negative influence a city may experience in the circumstance, alternatively, the ability of transportation to maintain its function of satisfying travelers’ travel demand. After conducting analysis of various scenarios, we can obtain the proportion of trips which can be converted into active modes. We divided the resilient level into five groups: very low (0–20.0%), low (20.1–40.0%), medium (40.1–60.0%), high (60.1–80.0%), and very high (80.1–100.0%).

### 2.2. Division and Encoding of Traffic Analysis Zone (TAZ)

The division of TAZ in this paper adopts the method of rasterization. Rasterizing the research area to obtain unit square areas, we consider these unit areas as TAZs and then we encoded each unit area to complete division. However, this kind of division is not fixed. Instead, the area size of each TAZ is adjusted according to the various ridesharing strategies for further analysis. After the processing is completed, pieces of individual trips are capable of being mapped to each single unit area. The data required in this paper can only be datasets including information of OD. Of note, for the consideration of travel distance in each trip, this paper has made a simplification, only accounting for the straight-line distance of each segment, and the MPD value to be adopted in the following part is also based on such principle.

### 2.3. Characteristics of Ridesharing Strategies

The main distinction between ridesharing strategies lies in the difference between similar trips matching criteria. In this place, we defined a concept of similar trips (ST) as: trips with similar origin, destination, and departure time. Only when trips are all in a group of ST can they select to adopt ridesharing service. Obviously, to distinguish STs, we need two parameters, one spatial parameter is used to confirm similar origins and destinations, and one temporal parameter to confirm similar departure. The spatial parameters are reflected in the side length of the TAZs we divided. We segmented the time of one day into groups with the same time interval, and the temporal parameters correspond to different time intervals. Different trips are classified as ST when their origins and destinations are in the same TAZ and their departures are in the same time interval.

In addition to these two parameters, the merging coefficient of trips selecting ridesharing service also needs to be considered. We define the merging coefficient as M. M reflects the willingness of travelers to select the ridesharing service which is equal to the proportion of STs choosing this service. This coefficient involves travelers’ consideration of ridesharing, such as delay, subsidy, comfort, and so on. It should be noted that M used in this paper is not a constant value, but one that can be adjusted and controlled by managers under the scenario of restricting motor vehicle travel. Considering the vehicle seat restrictions, the maximum setting of M is 0.4, which means 10 STs can be merged into 4 at least.

**Figure 2:**
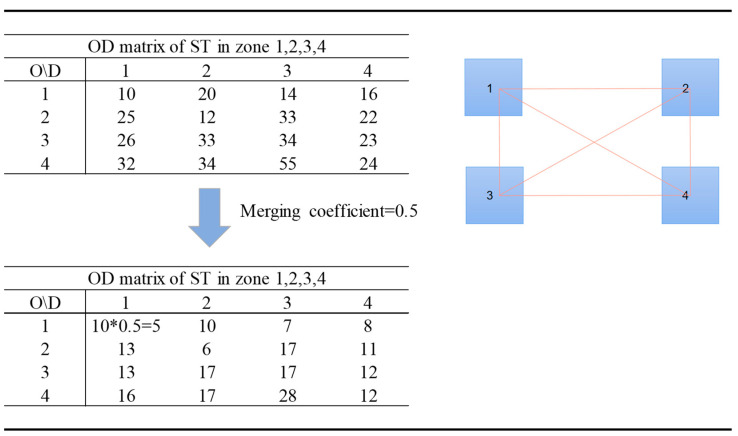
Adopting ridesharing service among TAZ 1,2,3,4.

In this context, all the ridesharing strategies are determined by the spatial parameters and temporal parameters. We have given a simple example to illustrate one of the strategies in Figure 2. Assuming that an area is composed of four TAZs (encoded as 1, 2, 3, 4) and each TAZ has the same size with a side length of 0.5 km. The OD matrix among the TAZs is given. Noting that the departure of trips in the OD matrix all in the same time group of 10 min intervals. OD travel matrix considering ridesharing service can be obtained via processing, as is shown in the left half of the figure.

### 2.4. Combination with Scenarios of MPD.

The approach to setting MPD scenarios depends on adjustment of MPD for cycling and walking. Regarding the adjustment of parameters in scenarios, we adopted the method to increase the MPD value of walking and cycling with incremental step length of 0.5 km to construct different scenarios. Likewise, for different ridesharing strategies, with consideration of realistic ridesharing service, we adopted the incremental step of 5 min for the time interval, and 0.5 km for side length.

In this paper, we have adopted a simple way of writing. The ridesharing strategies have two parameters, we used T and L to represent these two ones. For example, if the side length is 0.5 km and the interval of time division groups is 5 min, this strategy can be described as T5L0.5. T0L0 means no strategies are adopted. The labeling method for MPD scenarios is the same as [5]. We used W and C to represent MPD for walking and cycling. For instance, W1.5C2 means that in this scenario, the MPD value for walking is 1.5 km and the MPD value for cycling is 2 km.

### 2.5. Spatial Distribution of Resilience and Combination with Socioeconomic Statistics

After simulating different scenarios, the results obtained can be mapped to the research area to get the spatial distribution. As a result, we can see the resilient level of each region, so as to enable city managers to compare and draw some conclusions. Apart from this, it can also be combined with some socioeconomic statistics to analyze the relevance among them. In this paper, we adopted population distribution data to analyze the relationship with resilient level. Afterwards, the relevant conclusions can provide useful suggestions to policy and decision makers on the topic of urban transportation management and development [41]. This analysis will display the most critical areas and relatively weak areas for improving the overall resilience of the city so that the safety of transportation system is increased as well.

## 3. Applications

### 3.1. The Case Studies

In this paper, we used data from two cities in China: Shenzhen and Haikou as our analysis samples. Due to the limitations of data acquisition, data used in this paper only includes taxi trip data, which can reflect the characteristics of urban mobility to a certain extent. It should be noted that the contribution of this paper is to improve the framework of resilience assessment. When there is more complete data, the method proposed in this paper can be directly used for analysis without other changes. We chose these two cities for demonstration due to their distinctive characteristics of amount of trips, trip distribution, and city scale.

**Figure 3:**
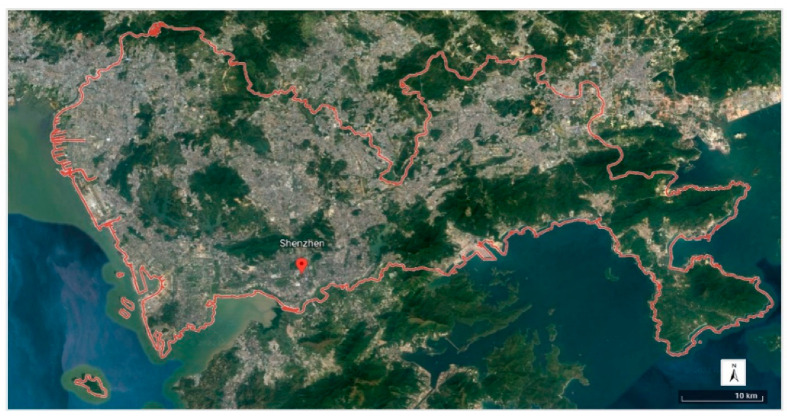
Region of Shenzhen, background Google Earth.

Shenzhen has one of the most developed road networks in China and has a high population density. The total area of Shenzhen is about 1997 square kilometers, and its permanent population is 10.63 million. It is divided into ten administrative districts, and the population density of each district varies greatly. As is shown in Figure 3, this is a port city approaching Hong Kong and it is with considerable number of ports and plenty of floating population China. This city has a well-developed internet industry and financial industry, which provides a plenty of related jobs.

**Figure 4:**
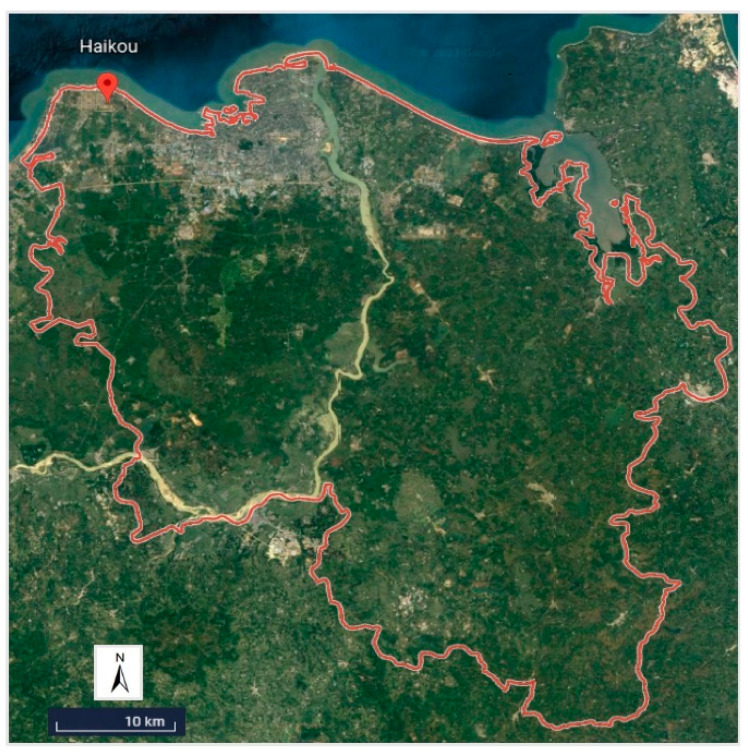
Region of Haikou, background Google Earth.

The characteristics of Haikou are quite different. The total area of Haikou City is about 2145 square kilometers, and the permanent population is about 2.27 million. Haikou has four administrative districts. As is shown in Figure 4, this city is also a port city. However, this city is a famous tourist city and its pillar industry is tourism.

The selected Shenzhen data is the itinerary data of taxis collected on 22 October 2013, which was a Tuesday. The selected Haikou data is the itinerary data of taxis collected on 16 May 2017. These two data sets consist of labels of latitude and longitude of OD and the departure of each trip. The data sets of the two selected cities are both a representative working day. Due to the limitation of data conditions, although we only have taxi data, taxi trips can reflect the urban mobility to a certain extent [35]. The sample size of the Shenzhen data is 443,815 and that of Haikou is 72,134. One thing we need to supplement here is that, due to limitations of data acquisition, we cannot make the analysis among datasets with a relatively short time gap. However, the most significant innovation of this study is to propose a framework to assess resilience when incorporating the impact of ridesharing strategies, and when more suitable datasets are available, no obvious change will need to be done to replace the original ones. This method only needs low-intensity data, and this will not be difficult to get for most city transportation managers. Apart from this, a considerable part of this study is to make comparative analysis between two cities, considering that there is a magnitude gap between Haikou and Shenzhen in terms of population, economy, and other social characteristics [42,43,44,45], although features of one city may change in several years, this will not obviously affect the comparison results between the two cities.

**Figure 5:**
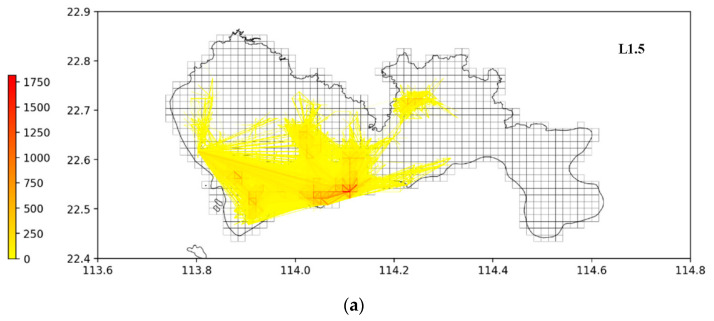

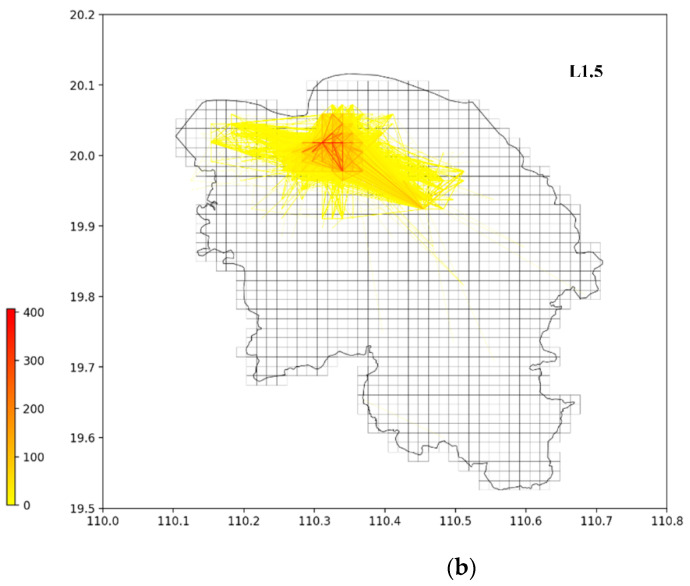
OD distribution map of two cities with side length of 1.5 km (**a**): Shenzhen (**b**): Haikou.

After simple processing, the OD trip information of the two data sets can correspond to the map. As shown in the Figure 5, we provide an example to rasterize the map and encode every unit as TAZ which has a side length of 1.5 km. Apart from this, we have distributed all the trips on the map, and the distribution of trips and itineraries is obviously unbalanced, regionalized, and centralized.

For consideration of temporal and spatial parameters cited in Section 2 in these two cities, we listed a total of 17 ridesharing strategies, which can be seen in Table 1. It is assumed that the M value of has been determined to be 0.5, which reflects a comparative high willingness to accept ridesharing services. As stated in the existing research [46,47], both passengers and drivers’ recognition of ridesharing service will be notably affected by waiting time and detour distance. Considering this, we set the maximum TAZ side length as 2 km and the maximum time interval as 20 min.

For all strategies above, we can calculate the total number of trips in the two cities after adopting the corresponding ones. We have presented the results in Table 2. Before adopting any ridesharing strategy, the total number of one-day trips in Shenzhen is 443,815 and that in Haikou is 72,134.

As a result of the ridesharing strategy, similar trips in daily trips will be merged, which means the amount of trips will be scaled down. In Table 2, by simulating strategies of ridesharing, the total number of trips can be reduced to a large extent. For example, in the context of L2T20, the number of trips in Shenzhen decreased by about 40% compared with L0T0, and that of Haikou can also reach about 35%. It is worth noting that changes in the number of trips seem to be more sensitive to the spatial parameters of the strategy. For example, by adopting the strategy from L0.5T5 to L2T5, due to the increase of qualified ST, the number of trips in Shenzhen and Haikou decreased by 30% and 22%. Correspondingly, from L0.5T5 to L0.5T20, the number of trips in Shenzhen and Haikou decreased by only about 3%. This shows that the spatial homogeneity of urban travel is more significant than the temporal homogeneity.

### 3.2. Resilience Level under Different Ridesharing Strategies and MPD Scenarios

In this part, based on the 17 ridesharing strategies that have been delineated, the MPD scenarios are further subdivided. Considering the characteristics of the Shenzhen and Haikou data used in this paper, an increase of 0.5 km is used as the iteration step of MPD. Regarding the characteristics of urban travel distance, the maximum walking MPD in Shenzhen is 4 km, and that of cycling is 40 km. A total of 456 scenarios are considered. The maximum MPD for walking in Haikou is 4 km and that for cycling is 21 km. A total of 228 scenarios are considered. The list of MPD scenarios is presented in Table 3.

After delineating all scenarios of MPD, different ridesharing strategies need to be added to further analyze the resilience level under these scenarios. Table 4 provides an example to evaluate resilient level of two cities by adopting the L1T10 strategy. It should be added that the MPD of riding and walking in Table 4 does not necessarily represent the value of real travel, but only to reflects the conditions that need to be met when different levels of resilience are achieved. When making a certain assessment, the MPD value should be determined in combination with the travel survey of the city. Initially when the MPD of riding and walking is 0 km, the resilience level of Haikou is 13.79% and that of Shenzhen is 13.84%, which means the proportion of trips that the adoption of L1T10 strategy reduces in the two cities. With the increase of MPD, the two cities show a similar changing pattern. The change of resilience can be divided into three stages. When the value of MPD is less than 3 km, the resilient level increases very rapidly (from C0W0 to C3W2), especially when the value of MPD is 2 km. When the value of MPD exceeds 3 km and less than 5.5 km, the growth of the resilience level is relatively gentle, but it is still significant, and the urban resilience reaches a high level. When the value of MPD exceeds 6 km, especially exceeding 10 km, the resilience level increases very slowly, and the resilience of the city reaches a very high level. This shows that the trips within the city are mostly concentrated in short- and medium-distance trips (less than 10 km). When the value of MPD reaches a certain degree, even if it continues to increase, the effect will not be significant.

In addition, it is worth noting that when the MPD of cycling is less than 6 km, the resilience level of Haikou is obviously higher than that of Haikou. This shows that Shenzhen is more affected by medium- and long-distance travel.

**Figure 6:**
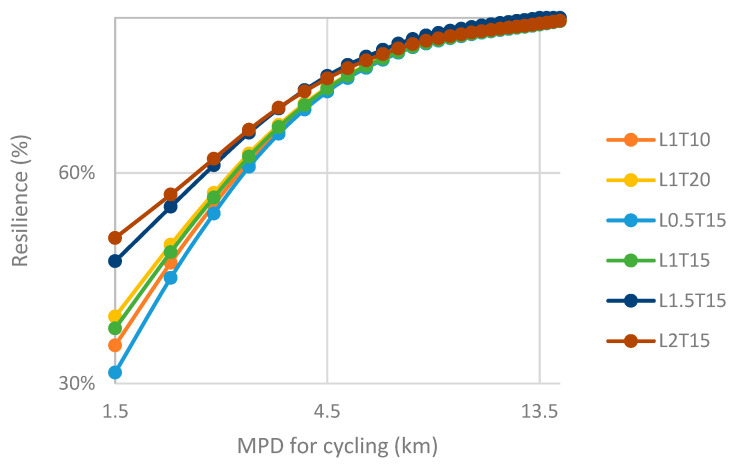
Comparison of resilience level under different ridesharing strategies, by adjusting the MPD for cycling (Haikou).

**Figure 7:**
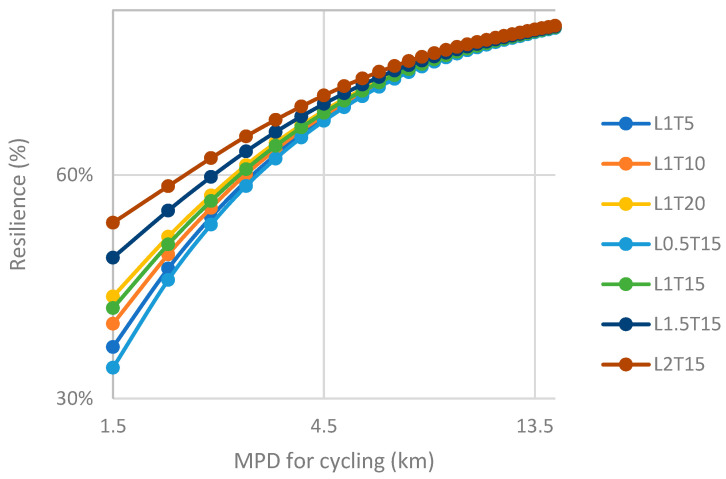
Comparison of resilience level under different ridesharing strategies, by adjusting the MPD for cycling (Shenzhen).

In order to show the impact of different ridesharing strategies on urban transportation resilience, we have compared and analyzed the result by adjusting the MPD for cycling in Figure 6 (Haikou) and Figure 7 (Shenzhen). The MPD for walking of these two cities is set to be 1.5 km.

In the figure, various line segments represent different ridesharing strategies. In this place, we selected several representative strategies from all 17 ones. Each node represents the MPD scenario with the value for cycling corresponding to the horizontal axis. The corresponding vertical axis is the resilience level in the scenario.

The entry resilience level of the strategy with the lowest performance (L0.5T15) in Haikou is about 32%, and the corresponding value of the strategy with the best performance (L2T15) is 50%. With the increase in MPD for cycling, the impact of changing ridesharing strategy on resilience becomes weaker. When the value of riding MPD is less than 3 km, the difference between various strategies is significant, while when the MPD of cycling exceeds 4.5 km, the difference in the resilience level between the different strategies can even be ignored, and it cannot be affected even if the parameters of ridesharing strategy are adjusted.

The entry resilience level of the strategy with the lowest performance (L0.5T15) in Shenzhen is about 35%, and the corresponding value of the strategy with the best performance (L2T15) is 55%. This data are obviously higher than the corresponding value of Haikou. Similar to Haikou, the impact of different strategies on resilience in Shenzhen tends to be insignificant with the increase of MPD for cycling, but this distinction almost disappears when it reaches about 6 km, which is greater than that of Haikou. This may be due to the larger proportion of short-distance trips in Shenzhen.

The average resilience level of Shenzhen under different strategies is higher than that of Haikou, which shows that ridesharing is more suitable under the travel characteristics of Shenzhen and has a broader development prospect in Shenzhen. Another observed finding is that the optimal strategy of Shenzhen and Haikou is (L2T15), while the worst strategy is (L0.5T15), which further shows that for urban mobility, the key to ridesharing strategy lies in its spatial parameters. This again verifies the previous conclusion.

### 3.3. Resilience Level Pattern under Various Merging Factors and Ridesharing Strategies

**Figure 8:**
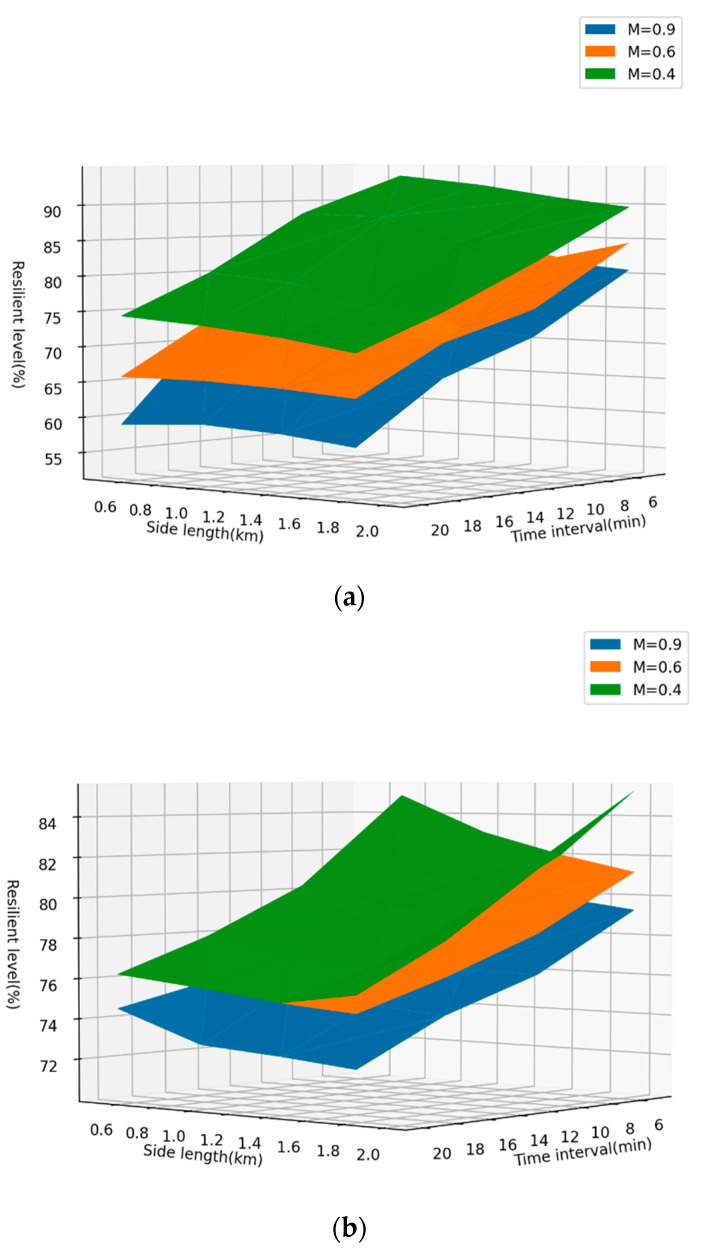
Comparison of resilience levels with various M under various ridesharing strategies (**a**). Shenzhen (**b**). Haikou.

In this section, this paper will show the changing patterns of the resilience levels of the two cities under different ridesharing strategies with various M, as shown in Figure 8.

The merging coefficient M reflects, to a certain extent, the willingness of travelers to accept ridesharing under the scenario of restricting motor vehicle travel, which depends on government subsidies, the comfort of ridesharing services, waiting time, and so on. In this paper, three Ms (0.4, 0.6, and 0.9) are selected to represent the three levels of willingness for ridesharing (high willingness, medium willingness, and low willingness). We set the MPD for cycling to be 4.5 km and the MPD for walking to be 1.5 km.

As can be seen from Figure 8, under the condition of low willingness for ridesharing, the resilience level of Haikou is higher than that of Shenzhen. However, with the improvement of willingness, the increment of the resilience level of Shenzhen will be significantly greater than that of Haikou. Apart from this, the resilience level distribution pattern of both Shenzhen and Haikou shows that when the willingness changes from medium to high, the gain of resilient level will be more obvious, compared with the change from low to medium. The analysis in this section will provide some references for urban traffic managers to promote ridesharing service.

### 3.4. Spatial Distribution of Resilience and Population Density

**Figure 9:**
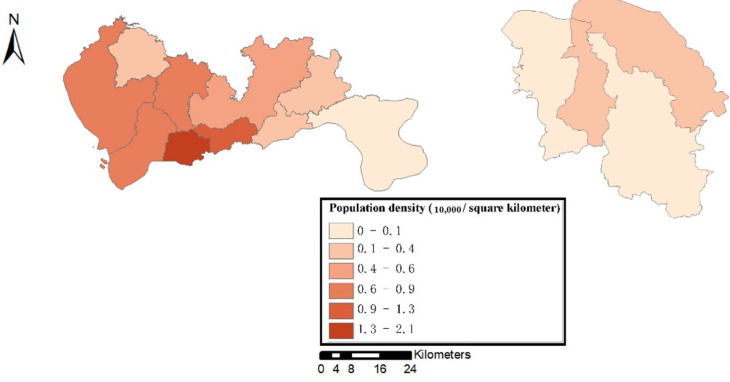
Spatial distribution of the population density (Left: Shenzhen Right: Haikou).

In this section, we explored the relationship between the spatial distribution of population based on administrative divisions and the level of resilience. To ensure legibility, we mapped the spatial distribution of population of these two cities in Figure 9 to identify the characteristics.

Shenzhen has ten administrative districts. The population density of Shenzhen is relatively large, and the density varies greatly among regions. Futian District, which has the highest population density, has reached the level of 21,000 people per square kilometer. In comparison, Haikou has a relatively sparse population distribution, and the population density levels among its four administrative regions are relatively similar. The Longhua District with the highest population density has only 2300 people per square kilometer.

**Figure 10:**
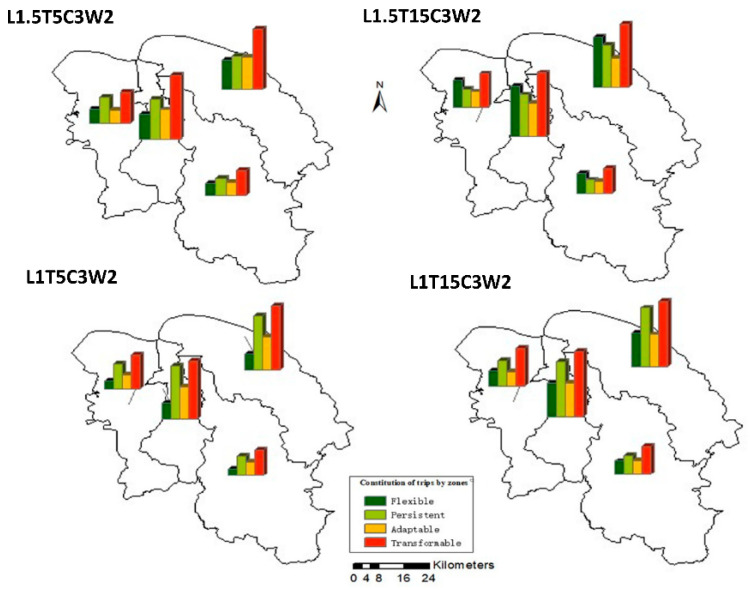
The distribution of trips in Haikou, under different ridesharing strategies.

Figure 10 shows the distribution of trips of each administrative region in Haikou under the selected four representative ridesharing strategies. The given MPD value of cycling is 3 km and that of walking is 2 km. It can be seen from the figure that with adopting different strategies, the composition of trips of each region is almost the same, and the changes of composition are also very similar. This may be because the population distribution of Haikou is relatively average and sparse, so the degree of time and space homogeneity of travel in each region is also very similar.

**Figure 11:**
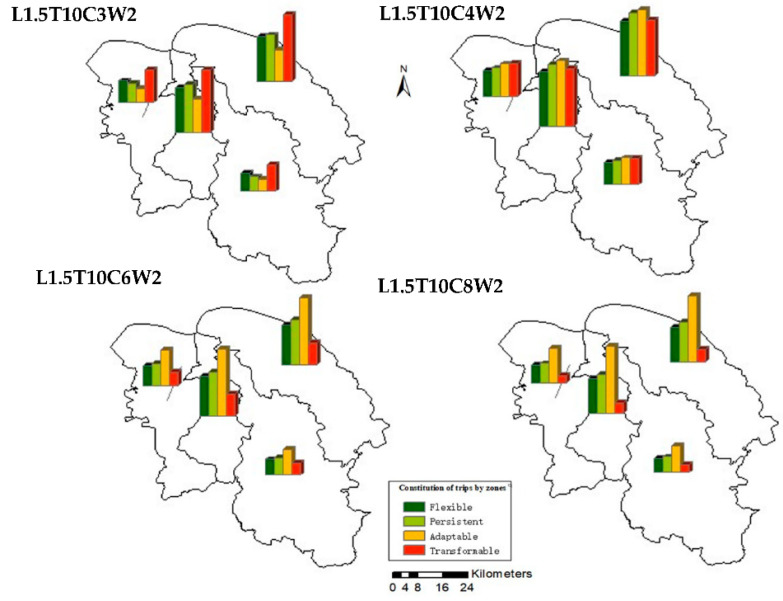
The distribution of trips in Haikou, with different MPD values for cycling.

Figure 11 shows the impact on the composition of trips of regions in Haikou by adjusting the MPD for cycling under the determined ridesharing strategy (L1.5T10). Similar to Figure 10, the proportions of resilient and non-resilient trips in different regions are very similar, and the impact of adjusting MPD for cycling is almost the same. This further reflects the homogeneity of characteristics of travel distances in various regions of Haikou.

**Figure 12:**
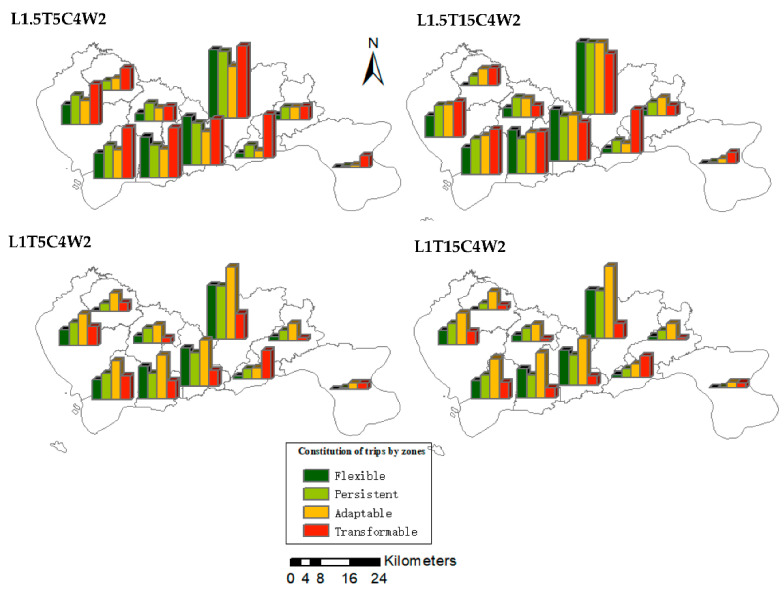
The distribution of trips in Shenzhen, under different ridesharing strategies.

Figure 12 shows the resilience level of Shenzhen’s administrative divisions under four ridesharing strategies. It can be observed that in the southern administrative regions, where the population is relatively concentrated, the proportion of flexible travel and the level of resilience are significantly higher than other regions. Meanwhile, with the change of the adopted strategy, the proportion of resilient travel in these areas also increase more significantly. The findings may be due to the fact that the southern area, as the central area of Shenzhen, has a high demand for travel. This means that ridesharing services are easier to find potential target groups in these regions, so appropriate ridesharing strategies are more effective in improving the resilience of these regions. Some administrative regions in the north and east have significantly lower levels of resilience and are insensitive to changes under different ST scenarios. This may be owing to the fact that the density of trips in these areas is low, and the space and time homogeneity of trips is not significant.

**Figure 13:**
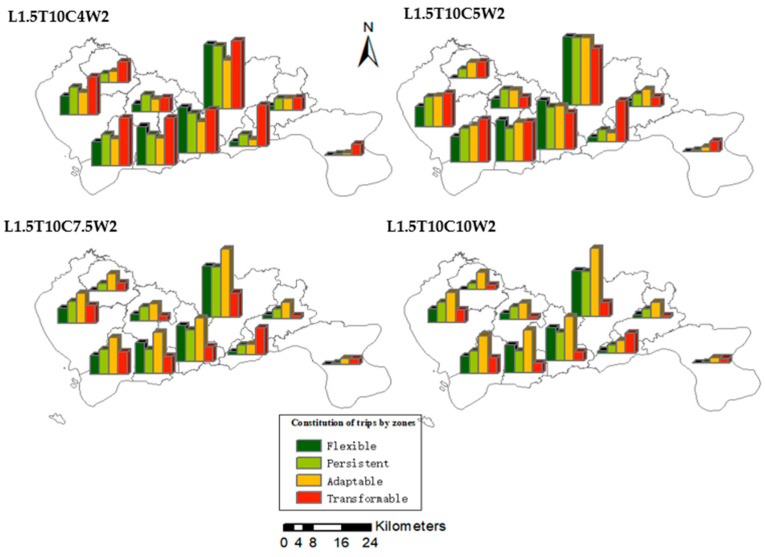
The distribution of trips in Shenzhen, with different MPD values for cycling.

Figure 13 shows the distribution and composition of trips in different regions of Shenzhen by adjusting the MPD for cycling under the determined ridesharing strategy (L1.5T10). It can be observed that with the increase in MPD for cycling, the resilience level of the administrative regions with higher population density increases more obviously. For example, when the MPD for cycling increases from 5 km to 7.5 km, the resilience of southern administrative region has been greatly improved, reaching a higher level of resilience, while the eastern and northern regions with lower population density have not changed much. This finding shows that the average travel distance in densely populated central regions is shorter, so the trips in these regions are easier to switch to active mode, while the travel distance in sparsely populated regions is generally longer, resulting in a minor proportion of resilient trips.

Therefore, we reach an interesting conclusion. For populous city, dense regions tend to have a higher level of resilience in the face of restricted use of motor vehicles. Taking some measures for dense areas such as adopting suitable ridesharing strategies, increasing cycling facilities can more significantly improve the overall resilience of the city. For non-dense regions, they are relatively vulnerable to the impact of events, so it is necessary to focus on improving its connection with the central area of the city.

## 4. Conclusions and Limitations

This paper proposes a framework for evaluating the overall urban transportation resilience in the case that the use of cars may be restricted or threatened. The resilience index adopted is the proportion of car trips that can be reduced or converted into active modes. A major innovation of this paper is that the impact of ridesharing services is considered in the proposed framework. By determining similar trips with similar origin, destination and departure and integrating various ridesharing willingness parameters, we can get the amount of car trips that can be reduced by ridesharing services, and finally the impact of ridesharing services on urban transportation resilience. The framework established in this paper can be integrated with urban economic and social data. In this paper, the relationship between the resilience level of urban administrative divisions and the population distribution of those is explored.

Numerical examples of two cities in China are provided to show the feasibility of the proposed method. The analysis shows that under the condition that the MPD remains unchanged, ridesharing services can effectively improve the overall resilience level of the city under the condition of restricting car use, because they can greatly reduce the total demand for car trips. From the perspective of operators and policymakers, increasing the radiation range of vehicles in ridesharing service can effectively increase the amount of trips that meet the conditions of carpooling, thereby improving the contribution of ridesharing services to reducing car trips. However, the loose ridesharing matching strategy may lead to the decline of ridesharing willingness, which is also be an important factor affecting the effect of carpooling services. This paper also analyzes this. Therefore, the framework proposed in this paper shows the potential of providing reference for policy makers to formulate the optimal ridesharing service strategy, which also involves many other factors such as subsidy setting.

The comparative analysis of the two cities in this paper shows that the resilience level distribution in Shenzhen, which is a city with a larger and more concentrated population, is more unbalanced. In contrast, the resilience level distribution in a city with a sparse and average population is more even, such as Haikou. However, due to the various city sizes, the MPD demand of Shenzhen reaching a high resilience level is obviously higher than that of Haikou, indicating that long-distance travel accounts for a larger proportion in Shenzhen. These findings indicate that this framework can provide a valuable reference for policy makers to formulate policies and allocate traffic resources according to different regions under specific circumstances cited above.

The framework proposed in this paper takes into account the impact of ridesharing services by considering the impact of MPD, which is different from existing research. The supplement makes this framework more universal. The data requirement of this framework is OD data, which is not difficult to obtain with current easier access to transportation big data (for example, mobile phone signaling data, RFID data, etc.), which shows the potential to be widely used in cities of various sizes. In addition, this framework also shows the potential of analyzing urban travel behavior characteristics in combination with other social and economic data.

Nevertheless, this paper still has some shortcomings. Firstly, this paper merely used the OD data of taxis, the future research should obtain more complete urban overall motor vehicle trips data as far as possible. In addition, in order to simplify the determination of the merging coefficient, this paper only uses a rough value, which lacks more detailed research. Therefore, in this paper only time and spatial factors are considered in the consideration of ridesharing, and more social and demographic factors can be added in future research, such as in the division of TAZ. Finally, this study focuses on the travelers with similar origin, destination, and departure, while we do not consider the circumstance that travelers may only share partial trips in the ridesharing service. Future research can consider this to enrich the hypothesis of this paper.

## Figures and Tables

**Table 1:** List of ridesharing strategies.

	Time Interval (min)				
Side Length of TAZ (km)	0	5	10	15	20
0	1	-	-	-	-
0.5	-	2	3	4	5
1	-	6	7	8	9
1.5	-	10	11	12	13
2	-	14	15	16	17

**Table 2:** Amount of Shenzhen (Haikou) trips after adopting ridesharing strategies.

	Time Interval (min)				
Side Length of TAZ (km)	0	5	10	15	20
0	443,815 (72,134)	-	-	-	-
0.5	-	432,980 (65,332)	426,625 (63,323)	421,329 (62,659)	416,734 (61,402)
1	-	403,008 (60,311)	382,376 (57,499)	368,574 (56,362)	358,155 (54,987)
1.5	-	346,105 (54,664)	321,405 (53,113)	307,347 (51,453)	297,639 (48,437)
2	-	304,129 (47,482)	281,348 (46,994)	269,219 (45,132)	261,081 (43,169)

**Table 3:** List of MPD Scenarios.

	MPD for Walking (km)								
MPD for Cycling (km)	0	0.5	1	1.5	2	2.5	3	3.5	4
0	1	-	-	-	-	-	-	-	-
0.5	-	2	-	-	-	-	-	-	-
1	-	-	3	-	-	-	-	-	-
1.5	-	-	-	4	-	-	-	-	-
2	-	-	-	5	6	-	-	-	-
2.5	-	-	-	7	8	9	-	-	-
3	-	-	-	10	11	12	13	-	-
3.5	-	-	-	14	15	16	17	18	-
21	-	-	-	223	224	225	226	227	228 *
39	-	-	-	439	440	441	442	443	444
39.5	-	-	-	445	446	447	448	449	450
40	-	-	-	451	452	453	454	455	456

* Last scenario tested for Haikou.

**Table 4:** Resilience level of various MPD Scenarios.

HaiKou				ShenZhen			
Scenario	MPD (km)			Scenario	MPD (km)		
	Cycling	Walking	Resilience (%)		Cycling	Walking	Resilience (%)
1	0	0	16.80%	1	0	0	13.84%
2	0.5	0.5	19.12%	2	0.5	0.5	16.90%
3	1	1	25.92%	3	1	1	26.91%
4	1.5	1.5	35.77%	4	1.5	1.5	37.83%
5	2	1.5	46.72%	5	2	1.5	46.90%
6	2	2	46.72%	6	2	2	46.90%
7	2.5	1.5	57.34%	7	2.5	1.5	54.19%
8	2.5	2	57.34%	8	2.5	2	54.19%
10	3	1.5	64.23%	10	3	1.5	60.14%
11	3	2	64.23%	11	3	2	60.14%
12	3	2.5	64.23%	12	3	2.5	60.14%
13	3	3	64.23%	13	3	3	60.14%
14	3.5	1.5	71.93%	14	3.5	1.5	64.90%
15	3.5	2	71.93%	15	3.5	2	64.90%
36	5	4	84.28%	36	5	4	75.04%
37	5.5	1.5	85.33%	37	5.5	1.5	77.47%
38	5.5	2	85.33%	38	5.5	2	77.47%
222	20.5	4	98.77%	450	39.5	4	99.91%
223	21	1.5	99.34%	451	40	1.5	99.92%
224	21	2	99.34%	452	40	2	99.92%
225	21	2.5	99.34%	453	40	2.5	99.92%
226	21	3	99.34%	454	40	3	99.92%
227	21	3.5	99.34%	455	40	3.5	99.92%
228	21	4	99.34%	456	40	4	99.92%

## Data Availability

Not applicable.
